# The CircumVent Project: a CPAP/O_2_ helmet solution for non-invasive ventilation using an implementation research framework

**DOI:** 10.1186/s43058-021-00193-y

**Published:** 2021-08-26

**Authors:** Aimalohi A. Ahonkhai, Adesola Z. Musa, André A. Fenton, Muktar H. Aliyu, Igho Ofotokun, Alex Hornstein, Baba M. Musa, Nnamdi Nwosu, Ifeoma Ulasi, Samuel Ajayi, Catherine Falade, Adedamola Dada, Aliyu Abdu, Mogaji Sunday, Adenike Odewabi, Muyiwa K. Rotimi, Onome Ogueh, Alan Steinbach, Gbenga Ogedegbe, Babatunde L. Salako, Oliver C. Ezechi

**Affiliations:** 1 Vanderbilt Institute for Global Health, Vanderbilt University Medical Center, Nashville, Tennessee USA; 2 Nigerian Institute of Medical Research, Lagos, Nigeria; 3 Center for Neural Science and Neuroscience Institute, NYU Langone Medical Center, New York, USA; 4 Neurobiology of Cognition Laboratory, Center for Neural Science and Neuroscience Institute, NYU Langone Medical Center, NY New York, USA; 5 Emory University, Atlanta, GA USA; 6 Ventilator Project, LLC, Boston, USA; 7 Aminu Kano Teaching Hospital, Kano, Nigeria; 8 University of Nigeria Teaching Hospital, Enugu, Nigeria; 9 University College Hospital, Ibadan, Nigeria; 10 Federal Medical Centre, Ebute Metta, Lagos, Nigeria; 11 Lagos University Teaching Hospital, Lagos, Nigeria; 12 Federal Medical Centre, Abeokuta, Nigeria; 13 Delta State University Teaching Hospital, Oghara, Nigeria; 14 Jefferson Health-Jefferson Torresdale Hospital, Philadelphia, USA; 15 Institute for Excellence in Health Equity, NYU Grossman School of Medicine, New York, NY USA

**Keywords:** SARS-CoV-2, COVID-19 infection, Nigeria, Non-invasive ventilation, Implementation science

## Abstract

**Background:**

Acute respiratory failure, a major cause of death in COVID-19, is managed with high-flow oxygen therapy via invasive mechanical ventilation. In resource-limited settings like Nigeria, the shortage of ventilators and oxygen supply makes this option challenging. Evidence-based non-invasive alternatives to mechanical ventilation such as the use of continuous positive airway pressure (CPAP) devices exist, but there have been concerns that non-invasive ventilation may expose healthcare workers to infection from aerosolized dispersion of SARS-CoV-2. We propose to evaluate the feasibility, adaptability and acceptability of a CPAP/O_2_ helmet solution for non-invasive ventilation among patients with COVID-19 and health workers in eight COVID-19 treatment and isolation centers in Nigeria.

**Methods:**

The study will occur in 4 stages: (1) convene a Steering Committee of key stakeholders and recruit implementation sites; (2) use the integrated Promoting Action on Research Implementation in Health Services (i-PARiHS) framework to guide a needs assessment of treatment centers’ capacity to use high-flow oxygen therapy to treat COVID-19 patients and utilize the findings to develop an implementation strategy for the use of a CPAP/O_2_ helmet solution; (3) build infrastructure to support training and data monitoring processes and to develop implementation protocols to evaluate the adaptability of the strategy for the use of the CPAP/O_2_ helmet; and (4) train health workers, distribute a CPAP/O_2_ helmet solution for non-invasive ventilation, pilot test the implementation strategy, and assess feasibility of its use and acceptability that includes monitoring altered risk of SARS-CoV-2 infection among healthcare workers.

**Discussion:**

The CPAP/O_2_ helmet solution for non-invasive ventilation in Nigeria can serve as a scalable model for resource-poor countries, and beyond the COVID-19 pandemic, has the potential to be deployed for the treatment of pneumonia and other respiratory diseases.

**Trial registration:**

NCT04929691. Registered June 18, 2021—retrospectively registered, https://clinicaltrials.gov/ct2/show/NCT04929691

Contributions to the literature

- Acute respiratory failure is a major cause of death in severe coronavirus disease 2019 (COVID-19).
- Evidence-based non-invasive alternatives to mechanical ventilation are needed to manage this condition.
- This paper describes a protocol to evaluate the feasibility, adaptability, and acceptability of a CPAP/O_2_ helmet solution for non-invasive ventilation among patients with COVID-19 and health workers in COVID-19 treatment centers in Nigeria.

## Background

The coronavirus disease 2019 (COVID-19) pandemic continues to surge across the globe, with more than 175 million persons infected with SARS-CoV-2 and causing over 2.6 million deaths, mostly due to acute respiratory failure [1]. With more than 89,000 deaths across sub-Saharan Africa, evidence-based just-in-time strategies that address acute shortage of ventilators and oxygen supply are in dire need, especially in countries like Nigeria, which accounts for over 20% of Africa’s population. Fortunately, the use of continuous positive airway pressure (CPAP) devices for non-invasive ventilation is an evidence-based alternative to mechanical ventilation [2–5]. CPAP and bilevel positive airway pressure (BiPAP) devices have been used in the management of patients with COVID-19 [6] and have inherent advantages, especially when intensive care resources are unavailable. CPAP and BiPAP machines can maintain positive end-expiratory pressure (PEEP) at 25 cmH_2_O and up to 50 l/min for FiO_2_ up to 95% with an appropriate patient circuit using supplemental oxygen flow [7]. When used with an oxygen helmet (O_2_ helmet), non-invasive ventilation (i) protects the treatment environment and healthcare workers from dispersion of the virus, (ii) improves outcomes [8], and (iii) reduces the need for mechanical ventilation [9]. CPAP devices can also run on 12 V DC, making them adaptable to battery use with solar-charging capability, an important consideration for low-income countries. CPAP devices are affordable, costing about 300 USD (1% the cost of a mechanical ventilator) and can readily be sourced from the approximately 3 million devices that are unused in U.S. homes to circumvent supply chain limits.

While randomized clinical trials have demonstrated the effectiveness of non-invasive ventilation (NIV) with BiPAP/O_2_ helmet use over face masks in reducing the need for endotracheal ventilation treatment of ARDS [10], CPAP/O_2_ helmet use has not been evaluated in low-resource settings like Nigeria, where mechanical ventilation is in limited supply, and oxygen as well as continuous electrical power supply is erratic. In response to this need, we established the CircumVent Project (www.circumventproject.com), a group of engineers, scientists, and clinicians, to develop a strategy to reduce the need for invasive mechanical ventilation and provide cost-effective alternative approaches for NIV in low- and middle-income countries (LMICs). The CircumVent Project will evaluate the feasibility of the CPAP/ O_2_ helmet solution using an implementation research framework that permits the exploration of the environment, the policy gaps, and the practice capacity for this non-invasive ventilation option in Nigeria. In this article, we describe the CircumVent Project as a potentially scalable model that can be implemented for large-scale deployment of CPAP/O_2_ helmets for non-invasive ventilation in Nigeria and other LMICs.

## Methods

### Study setting

The study will be conducted in eight COVID-19 treatment and isolation centers in Nigeria. The sites were selected based on availability of COVID-19 treatment services, geographic spread and patient volume. The eight sites are as follows: Aminu Kano Teaching Hospital; Delta State University Teaching Hospital; Federal Medical Centre, Abeokuta; Federal Medical Centre, Ebute Metta, Lagos; Lagos University Teaching Hospital; University College Hospital, Ibadan; University of Nigeria Teaching Hospital; and Alex Ekwueme Federal University Teaching Hospital.

### Study aims

1: Convene a Steering Committee of key stakeholders and recruit implementation sites to provide oversight for the development of processes for communication, decision-making, coordination, and distribution of CPAP/O_2_ helmet solutions for non-invasive ventilation at the eight COVID-19 treatment and isolation centers.

2: Use the integrated Promoting Action on Research Implementation in Health Services (i-PARiHS) framework to guide needs assessment, perform stakeholder analysis of the treatment centers’ capacity to use high-flow oxygen therapy to treat COVID-19 patients, and use the findings to develop a context-specific, practice facilitation strategy to implement the CPAP/O_2_ helmet solution for non-invasive ventilation.

3: Build infrastructure to support training and data monitoring processes and develop implementation protocols to evaluate the adaptability of the strategy for implementing use of CPAP/O_2_ helmets for non-invasive ventilation.

4: (a) Train healthcare workers; distribute a CPAP/O_2_ helmet solution for non-invasive ventilation, pilot test the practice facilitation [implementation] strategy, and assess feasibility of its use and acceptability; (b) estimate the altered risk of SARS-CoV-2 infection among healthcare workers associated with the use of CPAP/O_2_ helmets for non-invasive ventilation.

### Study design

The study will be performed in four phases: (1) assessment of the implementation climate (supply chain, distribution capacity, training); (2) deployment of CPAP/O_2_ helmet kits to each of the eight COVID-19 treatment centers in Nigeria and Training-of-Trainers (physicians) on the use of the CPAP/O_2_ helmet for the management of acute respiratory distress. The trainers will subsequently train non-physician personnel at their facilities on the use of the equipment; (3) assessment of acceptability of the CPAP/O_2_ helmets by physicians involved in the management of COVID-19 patients, and (4) assessment of change in the prevalence of COVID-19 infection among health care workers due to use of CPAP/O_2_ helmets for respiratory therapy.

### Conceptual framework

We will evaluate the feasibility of a CPAP/O_2_ helmet solution for non-invasive ventilation using the i-PARiHS framework. The core constructs of the framework include *facilitation*, *innovation*, *recipients,* and *context*, with *implementation facilitation* as the active element that integrates the other constructs. i-PARiHS posits that successful implementation of a program is specified in terms of the achievement of its goals, which results from facilitation of an innovation with the recipients and their context (local, organizational and health system) in which the program is being implemented [11]. i-PARiHS will guide the planning of the proposed study in 4 steps: *practice capacity assessment*, *training*, *distribution*, and *user testing.* Because scale up of an evidence-based program requires expertise to facilitate multilevel system changes [12], and LMICs often lack such expertise, we will use i-PARiHS to explore factors and support systems required for the successful use of CPAP/O_2_ helmets for non-invasive ventilation at the eight COVID-19 treatment centers in Nigeria.

### Study procedures

#### Treatment protocol for the CPAP/O_2_ helmet system

**Fig. 1:**
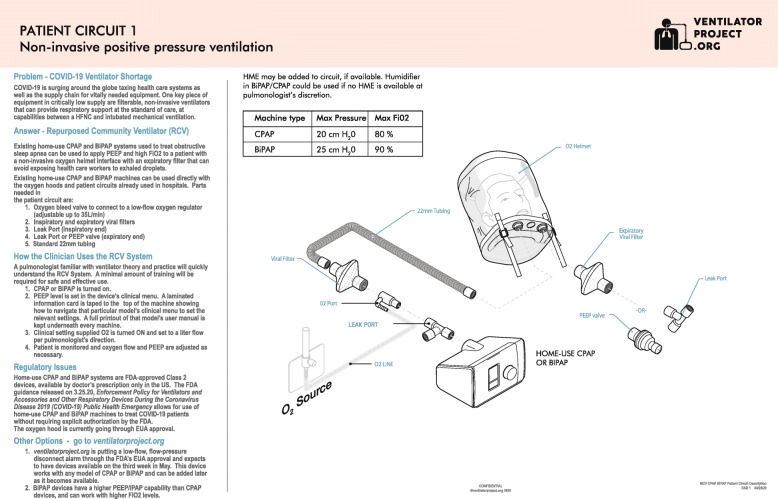
Patient circuit 1. Non-invasive positive pressure ventilation

The treatment protocol was designed in line with World Health Organization (WHO) and Nigerian national COVID-19 treatment guidelines. The patient interface is a low-cost Subsalve (North Kingstown, RI) oxygen helmet that can protect healthcare workers from viral exposure when used with disposable inspiratory and expiratory 22-mm viral filters (Fig. 1). The tubing and helmet can be sterilized in a variety of ways [13], including using low-cost steramine that is proven effective against SARS-CoV-2. The viral filters will prevent aerosolizing the SARS-CoV-2 virus and infecting healthcare providers for COVID-19 patients. Importantly, the oxygen helmet solves a problem that has contributed to the intubation of patients for mechanical ventilation, when NIV was also a feasible treatment [14–16].

#### Convening of Steering Committee (specific aim 1)

**Fig. 2:**
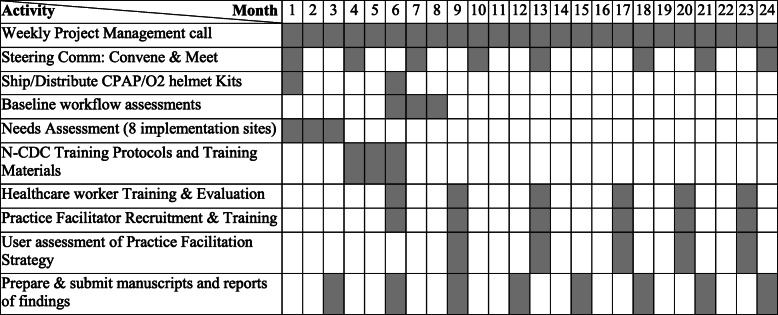
Timeline of project activities

The membership of the Steering Committee will be drawn from the Presidential Task Force on COVID-19; the Nigeria Ministry of Health, Directors of the COVID-19 Treatment and Isolation Centers, and the Nigeria Centre for Disease Control (NCDC). The Steering Committee will (1) identify the capacity of the eight COVID-19 treatment centers to use the CPAP/O_2_ helmet solution for non-invasive ventilation through stakeholder engagement, and (2) develop a context-specific practice facilitation strategy for use of the CPAP/O_2_ helmet kits in local hospitals. The Steering Committee will meet every 3 months (Fig. 2).

#### Implementation Committee

The Implementation Committee is comprised of medical directors/representatives of five of the eight COVID-19 isolation and treatment centers in Nigeria. The committee oversees implementation of the project including (a) identifying practice capacity of all eight COVID-19 treatment centers, (b) establishing a manual of procedures and data collection processes, (c) finalizing plans for program evaluation, and (d) testing the feasibility and acceptability of using the CPAP/O_2_ helmet solutions for NIV in COVID-19 patients. The Implementation Committee has been meeting weekly since June 2020 and since January 2021, meeting monthly.

#### Needs assessment (specific aim 2)

The structured needs assessment will explore treatment procedures for management of acute respiratory failure at the COVID-19 treatment centers, document staffing, staff characteristics (professional designation, training, experience), workload, facility characteristics (infrastructure, relevant supplies, oxygen supply), and service organization. The Steering Committee will employ findings from the needs assessment to refine all metrics to reflect usability of the measures and maintain scientific rigor.

We will use qualitative interviews and domains of i-PARiHS, to understand the context, barriers, and facilitators of implementing the CPAP/O_2_ helmet solution for non-invasive ventilation and contextual features of the COVID-19 treatment centers that will enable its implementation. These interviews will explore the following: (1) evidence of the likely fit of the treatment centers for use of the CPAP/O_2_ helmet solution for non-invasive ventilation; (2) the recipients and key stakeholders likely to be involved with implementing the use of a CPAP/O_2_ helmet solution for non-invasive ventilation in management of acute respiratory failure in COVID-19 patients, with a focus on the barriers that may influence its implementation; (3) context, resources and leadership support likely to influence acceptability and adaptability of the CPAP/O2 helmet solution for non-invasive ventilation; and (4) facilitation and the factors likely to enable recipients’ acceptability of the CPAP/O_2_ helmet solution for non-invasive ventilation.

Data will be collected from 16 physicians attending the CircumVent training who were selected from the participating health facilities. An interview guide will be developed, piloted, and used for the interviews. Participants will be contacted to schedule a convenient time for their interviews. Semi-structured interviews will be conducted in the English language. The opened-ended questions will allow the participants to express their opinions and share their experiences in the use of the CircumVent equipment, and suggestfactors that could promote or inhibit the use of the equipment. Prompts will be used to encourage participants to provide insight. Each interview will be audio-recorded. Each qualitative interview will last about 45 min. These data will inform development of the context-specific practice facilitation strategy.

#### Building infrastructure to support training and data monitoring processes and developing implementation protocols to evaluate adaptability (specific aim 3)

CircumVent leadership at the Nigeria Institute of Medical Research (NIMR) will lead this effort with guidance from the Implementation Committee. Proposed training and protocols will be developed based on the medical device training manuals from the manufacturer and adapted to the context of Nigeria. The Implementation Committee will apply needs assessment findings to the adaptation of existing protocols and refine existing materials. The Implementation Committee will also work with the NCDC to convene a cadre of trainers, whose sole purpose is to train health workers on the appropriate use of the CPAP/O_2_ helmets. These trainers will serve as coaches and provide support, knowledge exchange, and performance feedback to health workers using the CPAP/O_2_ helmets. Products from this phase will include training manuals, curricula, and standard operating procedures for the practice facilitation strategy and the use of the CPAP/O_2_ helmet kits.

#### Recruitment of implementation sites, health worker training, CPAP/O_2_ helmet distribution, pilot testing of practice facilitation strategy, and assessment of feasibility and acceptability (specific aim 4 a).

All eight COVID-19 treatment centers will be recruited for the proposed study.

*Training of health workers to use the CPAP/O*_*2*_ *helmets for non-invasive ventilation in COVID-19 patients*. Health workers will be trained on the components of the CPAP/O_2_ helmets, including the treatment, operation and cleaning protocols. The initial training will occur over 2 days, followed by a 1-day step-down and booster training sessions every 4 months over 1 year. Day 1 will focus on familiarizing workers with the CPAP device settings, understand and interpret oximeter readings, and general device maintenance. Day 2 will focus on training about the patient circuit, the logic of the tubing, and how to interface and work with the oxygen helmet, especially fitting it comfortably and effectively. The healthcare workers will have the opportunity to practice acquired skills via role playing and modeling with feedback, as well as review of the training materials.

*Training evaluation*. A baseline assessment will be done to evaluate the knowledge of the physician on the management of ARDS using the CPAP/O_2_ helmet. After the 2 days training, a post-training assessment will be done to evaluate the uptake of knowledge provided. The percentage improvement in knowledge will be used to determine the degree of uptake.

*Distribution of devices*. The Steering Committee will coordinate the distribution of devices using the feedback received from the stakeholder engagement. This distribution will consist of the following steps: (1) purchasing and transporting the CPAP/O_2_ helmet kits to Nigeria; (2) working with NIMR and the Nigerian Ministry of Health for the clearance of the devices through customs, their secure storage in Nigeria, and developing the protocol for disbursing the CPAP/O_2_ helmets to the treatment sites; and (3) NIMR will disburse the kits to the treatment centers and monitor their use.

*Practice facilitation strategy*. A practice facilitation strategy will be tailored to the needs of the eight treatment centers. Key stakeholders in the healthcare system (physicians, respiratory therapists, ICU doctors and ICU nurses) will take part in user testing of the developed practice facilitation strategy. For this purpose, the health workers who are expected to implement the use of the CPAP/O_2_ helmet solution for non-invasive ventilation will respond to a series of questions to explore the aspects of the devices and their implementation that are difficult to understand and probe their comprehension of the nature of the challenges.

*Training of practice facilitators using a train-the-trainer model*. After recruitment of practice facilitators, a “kick off” learning session will review components of the CPAP/O2 helmet solution for non-invasive ventilation and its protocol, quality improvement methods, the study timeline, and evaluation plan with the practice facilitators. Following this session, the key investigators and a pulmonologist will conduct a 2-day [train-the-trainer] training with them, followed by 1-day booster session after 4 months during the project implementation. The training will cover three core competencies of practice facilitation: data use to drive improvement, interpersonal skills, and quality improvement [17]. The training will help the facilitators achieve competencies in practice facilitation techniques such as effective communication skills, problem solving, promoting teamwork, consensus building, and goal setting. The training will combine didactic sessions with case-based learning, with booster sessions. The facilitators will serve as coaches, provide support, knowledge exchange, and performance feedback to the treatment centers.

### Is the use of the CPAP/oxygen helmet associated with a greater risk of COVID-19 infection among healthcare workers? (specific aim 4 b)

Healthcare personnel are at increased risk of acquiring COVID-19 infection [18]. Studies of other respiratory pathogens have documented increased transmission risk associated with aerosol generating procedures (AGP), many of which can generate both large droplets and small particle aerosols. Non-invasive ventilation is considered an AGP, but it is postulated that helmet ventilation may be associated with less risk of transmission of respiratory pathogens via aerosolization given containment in the helmet and negligible air dispersion.

A case-control study design will be implemented. We will enroll clinical and environmental hospital staff who have exposure to SARS-CoV-2 via provision of direct patient care or environmental care at the eight CircumVent study sites. Consenting and eligible health care workers will complete baseline testing for SARS-CoV-2-specific antibodies and complete an exposure assessment form summarizing risk factors and potential COVID-19 exposures in the prior 1 month. All healthcare workers assessed at time 0 comprise the retrospective study cohort. Individuals who test negative for COVID-19 antibodies at time 0 will be invited to participate in the prospective study. They will be followed for up to 6 months during which they will complete monthly encounter forms to assess community and healthcare-related exposures and to document any PCR-confirmed diagnoses of SARS-CoV-2 infection. However, the study will be censored and exit interview and a second sample for SARS-CoV-2 antibodies will be obtained if and when COVID-19 vaccine becomes available at the designated COVID-19 treatment centers.

In the retrospective study, a case is defined by seropositivity to SARS-CoV-2-specific antibodies, and a control is defined by seronegativity to SARS-CoV-2-specific antibodies. In the prospective study, a case is defined by seropositivity to SARS-CoV-2-specific antibodies, or a clinical case of SARS-CoV-2 infection confirmed by PCR testing, and a control is defined by the absence of infection, either by SARS-CoV-2 PCR or SARS-CoV2 specific antibodies.

### Implementation outcomes

- *Feasibility* is the extent to which the CPAP/O2 helmet solution for non-invasive ventilation can be successfully used within the Nigeria setting. We will employ mixed methods to measure feasibility from the patient and provider perspectives using in-depth-interviews (providers), structured case report forms assessing the fit/comfort of the helmet/CPAP device (patients), and any oxygen supply issues (for each case). We will also use the Feasibility of Intervention Measure (FIM), a 4-item instrument with a 5-point ordinal scale “completely disagree” to “completely agree” that takes less than 5 min to complete [19].
- *Adaptability* of the strategy for implementing the use of a CPAP/O2 helmet solution for non-invasive ventilation will be measured using a qualitative research approach, with focus on assessing the process of change, what providers want to change (ideal), what they need to have changed (essential), and what is adapted in the end (reality).
- *Acceptability* is the perception among implementation stakeholders that the CPAP/O_2_ helmet solution for non-invasive management of COVID-19 is agreeable, palatable, or satisfactory to patients and providers. We will assess acceptability of the use of the CPAP/O_2_ helmet solution from the patient and provider perspectives using in-depth-interviews (providers), structured case report forms assessing the fit/comfort of the helmet/CPAP device (patients), and any oxygen supply issues (for each case). We will also administer the Acceptability of Intervention Measure (AIM) to providers [19]. The instrument includes four items with a 5-point ordinal scale that ranges from “completely disagree” to “completely agree,”—higher scores indicate greater acceptability.

### Data analysis

#### Qualitative data analysis

Data collected will be transcribed verbatim after the interviews [20]. Data from the interviews will be transcribed and coded using open coding to develop an initial codebook by the qualitative research team. The codebook will include a list of question prompts, initial codes, and code meanings. Adopting Braun and Clarke’s six steps approach, codes will be reviewed, compared, analyzed, and sorted into categories to reflect consistent and overarching themes [21]. The team will develop a coding tree to derive themes until thematic saturation is attained [20–23].

#### Quantitative data analysis

Both the retrospective and nested prospective case-control studies will compare differences in exposures and risk factors observed between cases and controls using Chi-square or Fisher’s exact test. All tests will use 2-tailed probability, with *P* values < 0.05 considered statistically significant. Variables that are statistically significant will be included in forward logistic regression analysis to arrive at independent measures of association.

## Discussion

The COVID-19 pandemic will subside, but its effect on health systems will likely linger, particularly for African countries whose healthcare infrastructure is vulnerable. In this paper, we describe a non-invasive ventilation approach to management of COVID-19 in Nigeria using CPAP/O_2_ helmets. There are three key strengths in this study. First, this study uses a well-established implementation science framework (I-PARiHS) to assess the implementation climate, explore factors and support systems required for successful implementation of CPAP/O_2_ helmet solutions for non-invasive ventilation and to develop an evidence-based implementation strategy (practice facilitation) for the use of the helmets across eight COVID-19 treatment centers in Nigeria. Whereas extant literature in LMICs show that the uptake of evidence-based interventions is a desired implementation research outcome, there is limited evidence of interventions targeted at addressing care of COVID-19 patients with acute respiratory failure in Africa. Second, if successfully implemented, findings from this study will serve as pilot data for scaling up this strategy in other LMICs. The proposed project is in a position to collaborate with other groups to provide technology platforms to deliver training and support resources. Finally, the innovation that will be derived from the sourcing and using of these CPAP/O2 helmet solutions for non-invasive ventilation in Nigeria will serve as a model to other countries in Africa. The CPAP/O_2_ helmets can also be a major treatment for childhood pneumonia, the number one killer of children in low-income countries like Nigeria [24].

## Data Availability

The study PI and key investigators will have access to all data. Data access to other researchers will be considered on a case-by-case basis, upon reasonable request.

## Abbreviations

CPAP: Continuous positive airway pressure
COVID-19: Coronavirus disease 2019
SARS-CoV-2: Severe acute respiratory syndrome coronavirus type 2
NIMR: Nigeria Institute for Medical Research
NCDC: Nigeria Center for Disease Control
O_2_: Oxygen
i-PARiHS: Integrated Promoting Action on Research Implementation in Health Services
LMIC: Low- and middle-income countries
